# Prevalence and Risk Factors for Depression among Caregivers of Alzheimer's Disease Patients in Saudi Arabia

**DOI:** 10.1155/2018/2501835

**Published:** 2018-09-16

**Authors:** Mohammed Saeed Alqahtani, Ahmad Ayed Alshbriqe, Ahmed A. Awwadh, Turki Ali Alyami, Mohammed Saad Alshomrani, Adel Alhazzani

**Affiliations:** King Khalid University, Neurology-College of Medicine, Abha, Saudi Arabia

## Abstract

**Objectives:**

To identify prevalence and risk factors of depression among caregivers of Alzheimer' disease (AD) patients.

**Methods:**

In a cross-sectional study, 110 caregivers of AD patients participated in this study (51 males and 59 females). Patients data were obtained from patients' records at Aseer Central Hospital. Depression among caregivers was assessed by using the Hamilton Rating Scale for Depression.

**Results:**

The age of caregivers ranged from 17 to 85 years (Mean±SD: 41.1±14.0 years). Prevalence of depression among caregivers was 70%. Caregivers were mainly sons/daughters (69.1%) or spouses (11.8%). A total of 33 caregivers (30%) had mild depression while 44 (40%) had moderate depression. Prevalence of depression was significantly higher among caregivers of AD patients who were exposed to repeated falling down (p=0.003), but did not differ significantly among caregivers who were exposed to repeated pneumonia or getting lost. Caregivers' scores of depression positively correlated with duration of caregivers' daily stay with AD patients (r=0.272, p=0.004), but did not correlate significantly with either caregivers' age or patients' age. Moreover, caregivers' depression did not differ significantly according to their marital status, educational status, employment status, or monthly income.

**Conclusions:**

Prevalence of depression among AD caregiver is high. Risk factors for depression include patients' exposure to repeated falls and prolonged stay with patients. Therefore, psychiatric care should be provided to caregivers, home safety should be maintained to avoid falls, and several persons should interchangeably provide care to AD patients.

## 1. Introduction

The world's elderly population is rapidly growing [1]. This is inevitably accompanied by increase in age-related disorders, of which Alzheimer's disease (AD) is the most serious, mainly due to its economic and social burden [2].

Alzheimer's disease (AD) is a neurodegenerative disorder featuring gradually progressive cognitive and functional deficits as well as behavioral changes. Cognitive symptoms of AD most commonly include deficits in short-term memory, executive and visuospatial dysfunction, and praxis [3]. Moreover, AD patients' physical, mental, and social integrity are usually progressively compromised [4].

Prevalence of AD is rapidly increasing mainly because the proportion of elderly people is growing faster than any other age sector of the population worldwide [3]. Moreover, the AD is the leading cause of disability among people above 65 years old. Patients usually have reduced capacity to live independently and need increasingly complex care. With disease progression, the need for care and continued supervision increases. Therefore, the importance of the family is essential to provide care to the elderly. The changes that take place in the life of caregivers may cause physical and emotional burden, acute and chronic diseases, and financial deterioration, affecting all activities [5].

Family members caring for their loved ones often describe the caregiving experience as stressful and frustrating [6]. Moreover, the physical and emotional condition of the caregiver directly affects the quality of care provided to the Alzheimer's patient. Caregiver burden may give way to patient abuse, both physical and psychological, and even neglect of the patient [5].

This study aimed to identify prevalence and risk factors of depression among caregivers of Alzheimer patients.

## 2. Subjects and Methods

This study followed a cross-sectional design, which has been conducted during the period from August to November 2016.

Based on AD patients' data that were obtained from their hospital records at Aseer Central Hospital in Abha City, Aseer Region, Kingdom of Saudi Arabia, a total of 110 caregivers of AD patients (51 males and 59 females) were recruited into this study.

Sociodemographic data were collected from participants. Depression among caregivers was assessed by using the Hamilton Rating Scale for Depression. Severity of AD was considered according to disease duration, hospital admission, presence of infections (e.g., pneumonia), and other associated complications (e.g., repeated falls, bone fractures, and getting lost).

The ethical approval for conducting this study was obtained from the Ethical Committee in King Khalid University. The informed written consent of participants was obtained prior to their participation in this study.

Collected data were analyzed using the Statistical Package for Social Sciences (SPSS version 22). Descriptive statistics (i.e., frequency, percentage, mean, and standard deviation) were calculated. Chi-square test of significance was applied to determine the significance of differences in prevalence rates of depression among caregivers according to study variables. Fisher exact test was applied when more than 25% of expected values were less than 5. Pearson's correlation coefficient (r) was calculated between caregivers' depression scores and quantitative variables (i.e., their age and duration of care to AD patients). P-values less than 0.05 were considered as statistically significant.

## 3. Results

Table 1 shows that age of 56.4% of participant caregivers was 40 years or less, with a mean age (SD) of 41.1 (14) years. More than half of caregivers (53.6%) were females. Most of the caregivers (69.1%) were married, while 20% were single, 2.7% were divorced, and 8.2% were widowed. Participants were mainly university or secondary school educated (40% and 39.9%, respectively). Most participants were students or employed (49.1% and 40%, respectively). The monthly income of 44.5% of participants was 3000-6000 SR, while 20% had a monthly income of 6001-10000 SR.

Table 2 shows that most participants (69.1%) were sons/daughters to AD patients, while 11.8% were spouses, 6.4% were parents, and 2.7% were brothers/sisters. The most frequently witnessed complications affecting AD patients were pneumonia (60.9%), and being lost (60.9%), followed by repeated falls (51.8%) and fractures (18.2%).

**Figure 1:**
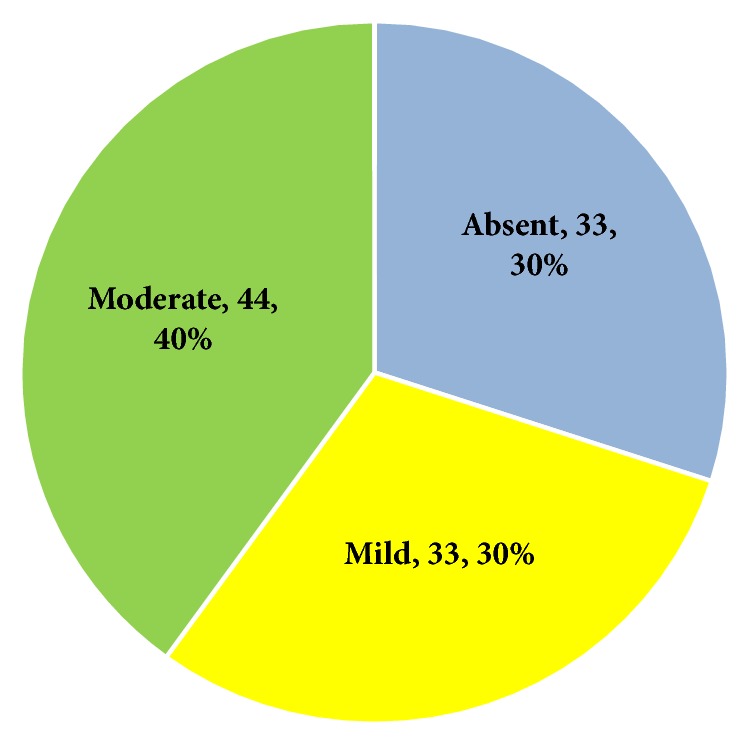
Grades of depression symptoms among caregivers of AD patients.

Figure 1 shows that prevalence of depression symptoms among caregivers was 70%. A total of 33 caregivers (30%) had mild depression, while 44 (40%) had moderate depression. None of the participant caregivers had severe depression.

Table 3 shows that prevalence of depression was highest among caregivers with lowest monthly income and lowest among caregivers with highest monthly income (p=0.037). However, prevalence rates among caregivers did not differ significantly according to other studied personal characteristics of caregivers.

Table 4 shows that prevalence of depression among caregivers was significantly higher when AD patients sustained repeated falls (p=0.011). However, the prevalence of depression among caregivers did not differ significantly according to other studied AD patients' characteristics.

Table 5 shows that caregivers' depression scores significantly and positively correlated with duration of caregiving (r=0.272, p=0.004), but not with their age.

Table 6 shows that disease duration of AD patients in this study was less than two years among 33.6% of AD patients, 2-3 years among 35.5% of AD patients, and more than three years among 30.9% of AD patients. More than two-thirds of AD patients (70%) were previously hospitalized. Pneumonia and getting lost were the most common complications among AD patients (60.9% for both), while repeated falls and bone fracture occurred in 50.9% and 18.2% of AD patients. Severity of caregivers' depression did not differ significantly according to patients' duration of disease, hospital admissions, being infected with pneumonia, exposure to bone fracture, and repeated exposure to falls or getting lost.

## 4. Discussion

Alzheimer's disease (AD) is a progressive neurodegenerative disease. As the disease progresses, patients become more cognitively impaired and experience a decrease in functionality [7]. This can be hard for caregivers and can considerably affect their mental health, family life, job, and finances [1]. Caring for the patient with dementia has been described as one of the most demanding situations encountered [8].

This study aimed to identify prevalence and risk factors of depression among caregivers of Alzheimer patients.

Results of this study showed that prevalence of depression symptoms among caregivers of AD patients was very high, i.e., 70%, 30% had mild depression symptoms, and 40% had moderate depression symptoms, while none had severe depression symptoms.

This finding is in accordance with that reported by Papastavrou et al., who noted that as much as 65% of caregivers do experience symptoms suggestive of depressive symptoms in the process of care [9].

Our finding is also in agreement with that of Ostojić et al. [10], who used the Hospital Anxiety and Depression Scale (HADS) to report that prevalence rate for anxiety and depression among caregivers of the AD was 56.7%, with 30% being borderline and 26.7% pathologic.

Lower prevalence rates for depression symptoms among AD patients' caregivers were reported by several other studies. Lowery et al. [11] reported a prevalence rate of 30% for depression symptoms among caregivers of dementia.

The systematic review of Sallim et al. [12] concluded an aggregate prevalence of 34% for depression among caregivers of the AD, which is far above the 5% prevalence of depression within the general population. They also noted that that prevalence of depression among caregivers of AD patients was remarkably higher than caregivers of patients with psychiatric diseases other than AD, e.g., schizophrenia (19.5%), reported by Thunyadee et al. [13] or among caregivers of patients with a physical illness, e.g., stroke (18.8%), reported by Atteih et al. [14].

These findings indicate that caregivers of AD patients are at high risk of depression. Shua-Haimet al. [8] stated that caregivers of AD patients experience depression symptoms during the course of the disease. Common associated risk factors for caregivers' depression include their social isolation, reduced control over their lives, fear of inadequacy, loss of their social relations, and their frequent lack of positive reinforcement.

Prevalence of depression among caregivers of the AD in the present study is higher than those reported by many studies in other countries. This finding can be explained by that caregivers for AD patients in the present study were mainly their sons/daughters (69.1%) with the strongest emotional relations toward their parents with the AD. Moreover, Abyad added a special character, that specially describes that the Middle Eastern Arab culture, which highly values the natural bonds of affection between all members of the family and the eldest members constitute a source of spiritual blessing as well as models of piety, religious faith, wisdom, and love. Therefore, any disease that affects an elderly member is usually negatively reflected upon all other members of the family [1].

The wide variations in reported rates for the prevalence of depression symptoms among caregivers of AD patients may be due to different diagnostic tools and different population characteristics.

Results of the present study showed that almost two-thirds of caregivers witnessed AD patients that they were caring for getting pneumonia or getting lost, while about half of them witnessed them repeatedly falling and some of the AD patients sustained fractures. Prevalence of depression among caregivers was significantly higher when AD patients sustained repeated falls. Moreover, caregivers' depression scores significantly correlated with duration of caregiving.

Volicer stated that the most common cause of hospitalization for AD patients is an infection, most often pneumonia. Risk of pneumonia is increased among AD patients who are confined to bed, have an associated debilitating neurologic disease, and are fed by a tube, those with swallowing difficulties and inability to take oral medications [15].

Horikawa et al. reported that falls are common among patients with the AD. They suggested that periventricular white matter lesions and the use of neuroleptics may be related to increased incidence of repeated falls in cases of theAD [16].

The Alzheimer's Association also stated that 60% of patients with AD eventually wander and get lost [17]. Tetewsky and Duffy stated that AD commonly causes patients to get lost even in familiar surroundings, which occurs partly because of visuospatial disorientation from parietooccipital involvement [18].

It is not astonishing that AD is commonly associated with several complications, and it is not surprising to find depression among their caregivers being significantly associated with any of these complications. Depression among caregivers may be further augmented with the addition of a complication to the frail person they are responsible to take care of. Moreover, it is not surprising to find more depression symptoms among those who provide care to AD patients for longer times.

Findings of the present study showed that personal characteristics of caregivers were not significantly associated with their depression symptoms. However, Levine reported that some of the independent factors that predicted caregiver depression were their younger age, having less education, low income, and prolonged duration of caregiving [19].

Apostolova concluded that the effect of the AD on individual patients and their families and caregivers is devastating. Unless a definite cure for the AD is reached, the incidence of the AD will continue to climb. Therefore, families, caretakers, the medical system, and society as a whole should continue to bear the heavy burden of this disease [3].

However, a definite cure for the AD is still a dream, better management of dementia care carries promising breakthroughs. Zhang et al. stated that, with the rapid advances in technology, information technology and its related innovations have been used in dementia care. An evidence based manual has been integrated into a smartphone application, which was programed using a new methodology. This application is meant to help both the patient living with dementia and their caregiver. The application comprises several subsections, i.e., information, videos, podcasts, mindfulness exercises, medications, daily questionnaire assessment, assessment questionnaire, records of questionnaire scores, and a photograph library and a journaling function [20].

Zhang et al. added that a unique visual pill tracker was included to enable AD caregivers to take a photo of the medications and log down the timing that their loved ones need to take the respective medications. Caregivers would be notified via a push notification of the right timing to administer the respective medications. Moreover, there are baseline questionnaires, e.g., the HADS, which help them to self-monitor their levels of anxiety and depression across a period of time [20].

The current study revealed that pneumonia and getting lost were the most common complications among AD patients. Moreover, different aspects of patients' AD severity were not significantly associated with severity of depression among their caregivers.

These findings are in accordance with those reported with Kalia [21], who noted that dysphagia and aspiration pneumonia are the two most serious medical conditions seen in AD patients. The main causes for pneumonia are reduced patient's level of consciousness, dysphagia, and loss of the gag reflex. Moreover, Pai and Lee [22] stated that getting lost behavior is highly prevalent in patients with AD, with an approximate 40% of patients reportedly experiencing it. However, the prevalence of getting lost increases to 70% in patients with severe AD [23].

In agreement with findings of the current study, several studies reported no significant association between severity of dementia and caregivers' depression [24, 25]. Välimäki et al. [26] explained the insignificant correlation between the AD patients' disease severity and their caregivers' degree of depression by the fact that caregivers of patients with dementia are already at the lowest level of health-related quality of life compared with the general population.

The present study provided further knowledge regarding the characteristics of AD caregivers who are at high risk of depression in the Saudi community. Therefore, communication between neurologists and family members who provide daily care for AD patients is essential for continued provision of optimal homecare to AD patients.

## 5. Conclusion

Symptoms of depression are highly prevalent among caregivers of AD patients. Risk factors for depression symptoms among caregivers include AD patients' exposure to repeated falls and prolonged stay with patients. Therefore, psychiatric care should be provided to caregivers, home safety should be maintained to avoid falls, and several persons should interchangeably provide care to AD patients.

## Figures and Tables

**Table 1:** Personal characteristics of study sample.

Characteristics	No.	%
Age groups		
(i) <40 years	62	56.4
(ii) 40+ years	48	43.6
(iii) Mean±SD (in years)	41.1±14.0	
Gender		
(i) Male	51	46.4
(ii) Female	59	53.6
Marital status		
(i) Single	22	20.0
(ii) Married	76	69.1
(iii) Divorced	3	2.7
(iv) Widowed	9	8.2
Educational status		
(i) Primary	21	19.1
(ii) Intermediate	11	10.0
(iii) Secondary	34	30.9
(iv) University	44	40.0
Employment status		
(i) Student	54	49.1
(ii) Employed	44	40.0
(iii) Unemployed/housewife	12	10.9
Monthly income		
(i) <3000 SR	18	16.4
(ii) 3000-6000	49	44.5
(iii) 6001-10000	22	20.0
(iv) >10000	21	19.1

**Table 2:** Characteristics of AD patients as stated by caregivers.

Characteristics	No.	%
Relation to AD patients		
(i) Spouse	13	11.8
(ii) Son/Daughter	76	69.1
(iii) Brother/Sister	3	2.7
(iv) Parent	7	6.4
(v) Other	11	10.0
Encountered complications among AD patients		
(i) Pneumonia	67	60.9
(ii) Getting lost	67	60.9
(iii) Repeated falls	57	51.8
(iv) Fractures	20	18.2

**Table 3:** Prevalence of depression among caregivers according to their personal characteristics.

	Absent (n=33)		Present (n=77)		P
Personal Characteristics	No.	%	No.	%	value
Age groups					
(i) <40 years	14	22.6	48	77.4	
(ii) 40+ years	19	39.6	29	60.4	0.054
Gender					
(i) Male	16	31.4	35	68.6	
(ii) Female	17	28.8	42	71.2	0.770
Marital status					
(i) Single	6	27.3	16	72.7	
(ii) Married	22	28.9	54	71.1	
(iii) Divorced	1	33.3	2	66.7	
(iv) Widowed	4	44.4	5	55.6	0.794
Educational status					
(i) Primary	7	33.3	14	66.7	
(ii) Intermediate	3	27.3	8	72.7	
(iii) Secondary	11	32.4	23	67.6	
(iv) University	12	27.3	32	72.7	0.941
Employment status					
(i) Student	17	31.5	37	68.5	
(ii) Employed	12	27.3	32	72.7	
(iii) Unemployed/housewife	4	33.3	8	66.7	0.871
Monthly income					
(i) <3000 SR	1	5.6	17	94.4	
(ii) 3000-6000	16	32.7	33	67.3	
(iii) 6001-10000	6	27.3	16	72.7	
(iv) >10000	10	47.6	11	52.4	0.037

**Table 4:** Prevalence of depression among caregivers according to characteristics of AD patients.

	Absent (n=33)		Present (n=77)		P
Characteristics	No.	%	No.	%	value
Relation to AD patients					
(i) Spouse	4	30.8	9	69.2	
(ii) Son/Daughter	21	27.6	55	72.4	
(iii) Brother/Sister	1	33.3	2	66.7	
(iv) Parent	1	14.3	6	85.7	
(v) Other	6	54.5	5	45.5	0.402
Complications among AD patients					
(i) Pneumonia					
(a) Present	20	29.9	47	70.1	
(b) Absent	13	30.2	30	69.8	0.966
(ii) Getting lost					
(a) Present	17	25.4	50	74.6	
(b) Absent	16	37.2	27	62.8	0.186
(iii) Repeated falls					
(a) Present	11	19.3	46	80.7	
(b) Absent	22	41.5	31	58.5	0.011
(iv) Fractures					
(a) Present	4	20.0	16	80.0	
(b) Absent	29	32.2	61	67.8	0.281

**Table 5:** Correlation coefficients (r) between caregivers' depression scores with their age and duration of caregiving.

Variables	Correlation coefficient	p-value
Age of caregiver	0.005	0.963
Duration of caregiving	0.272	0.004

**Table 6:** Relation between severity of caregivers' depression and severity of dementia.

Indicators of dementia severity	Absent (n=33)		Mild (n=33)		Severe (n=44)		Total (n=110)		P
	No.	%	No.	%	No.	%	No.	%	Value
Duration of disease									
(i) < 2 years	14	37.8	11	29.7	12	32.4	37	33.6	
(ii) 2-3 years	9	23.1	13	33.3	17	43.6	39	35.5	
(iii) > 3 years	10	29.4	9	26.5	15	44.1	34	30.9	0.650
Hospital admissions									
(i) No	9	27.3	14	42.4	10	30.3	33	30.0	
(ii) Yes	24	31.2	19	24.7	34	44.2	77	70.0	0.161
Pneumonia									
(i) No	13	30.2	14	32.6	16	37.2	43	39.1	
(ii) Yes	20	29.9	19	28.4	28	41.8	67	60.9	0.864
Repeated falls									
(i) Yes	12	21.4	16	28.6	28	50.0	56	50.9	
(ii) No	21	38.9	17	31.5	16	29.6	54	49.1	0.057
Bone fracture									
(i) Yes	4	20.0	6	30.0	10	50.0	20	18.2	
(ii) No	29	32.2	27	30.0	34	37.8	90	81.8	0.490
Getting lost									
(i) Yes	17	25.4	20	29.9	30	44.8	67	60.9	
(ii) No	16	37.2	13	30.2	14	32.6	43	39.1	0.333

## Data Availability

The data used to support the findings of this study are available from the corresponding author upon request.

## Abbreviations

AD: Alzheimer's disease
SPSS version 22: Statistical Package for Social Sciences
HADS: Hospital Anxiety and Depression Scale.

## Additional Points

*Advances in Knowledge (How the Article Contributes to This).* (i) Family members can play an essential role in caring for Alzheimer's disease (AD) patients. (ii) Caregivers' stress often leads to mental and physical ill-health. (iii) This study provides further knowledge regarding the characters of AD caregivers who are at high risk of depression in the Saudi community.* Application to Patient Care (How the Article Contributes to This).* Results of this study highlight the pressing need to extend communication between neurologists and family members who provide daily care for AD patients so as to enable them to continue provision of optimal patient care at home.
